# Bridging the Gaps in Dengue Control in Latin America: Multisectoral Strategies from an Expert Panel

**DOI:** 10.3390/vaccines14060488

**Published:** 2026-05-30

**Authors:** Carlos Espinal Tejada, Marisa Aizenberg, Zulma Cucunubá, Wilfrido Coronell Rodríguez, Judit Díaz Bazán, Fernando Ariel García Terrón, Gamaliel Gutiérrez, Alejandro Llanos Cuentas, Martín Casapia Morales, José Guadalupe Martínez Núñez, José Alejandro Mojica, Tomás Orduna, Victoria Pando-Robles, Mariana Rico-Restrepo, Jaime R. Torres, Mauricio Javier Vera Soto, Iván Darío Vélez

**Affiliations:** 1 Department of Global Health, Robert Stempe, Florida International University, Miami, FL 33174, USA; 2 Health Observatory, Faculty of Law, University of Buenos Aires, Buenos Aires C1121A6B, Argentina; marisaaizenberg@yahoo.com.ar; 3 Department of Clinical Epidemiology and Biostatistics, Faculty of Medicine, Pontificia Universidad Javeriana, Bogotá 110231, Colombia; 4 School of Medicine, Universidad de Cartagena, Cartagena 130001, Colombia; 5 Women in Global Health, Buenos Aires C1017AAC, Argentina; 6 AIDS Healthcare Foundation Global Public Health Institute for Latin America and the Caribbean, Mexico City 11590, Mexico; 7 Pan American Health Organization, Washington, DC 20037, USA; 8 Alexander von Humboldt Tropical Medicine Institute, Universidad Peruana Cayetano Heredia, Lima 15102, Peru; 9 Faculty of Human Medicine, National University of the Peruvian Amazon, Iquitos 16001, Peru; 10 Pediatrics and Infectious Diseases, National Network of Experts in Vector-Borne Diseases, Centro Nacional de Prevención y Control de Enfermedades (CENAPRECE), Ministry of Health, Mexico City 06720, Mexico; 11 Ministry of Health and Social Protection, Bogotá 111711, Colombia; 12 Francisco Javier Muñiz Infectious Diseases Hospital, Buenos Aires C1282AEN, Argentina; 13 Center for Research on Infectious Diseases (CISEI), National Institute of Public Health, Cuernavaca 62100, Mexico; 14 Americas Health Foundation, Bogota 110221, Colombia; 15 Tropical Medicine Institute, Universidad Central de Venezuela (UCV), Caracas 1050, Venezuela; 16 Program for the Study and Control of Tropical Diseases (PECET), University of Antioquia, Medellín 050010, Colombia

**Keywords:** dengue, strategies, vaccination, barriers, Latin America

## Abstract

In March 2025, the Americas Health Foundation (AHF) convened a regional meeting, in Bogotá, Colombia, of the Latin America Dengue Task Force, a multidisciplinary regional group of arbovirus specialists, to address the growing challenges posed by dengue in the region. The primary objective of the meeting was to analyze the regional dengue landscape, identify gaps and barriers that hinder a comprehensive approach, and develop a strategic blueprint for advancing control and prevention in Latin America. The Task Force conducted a thorough review of dengue-related issues, drawing on participants’ professional expertise in vaccination, prevention, diagnosis, treatment, vector control, environmental determinants, and regulatory and policy frameworks. Key barriers identified include underreporting, widespread insecticide resistance, structural weaknesses in the health system, fragmented surveillance, financial and political constraints, and socioeconomic drivers. Based on these findings, actionable recommendations were developed to optimize regional dengue surveillance, strengthen prevention and diagnosis strategies, and improve coordination among stakeholders. This narrative review summarizes the epidemiological context, presents expert guidance to overcome existing limitations, and outlines strategies to advance integrated dengue control in Latin America.

## 1. Introduction

Dengue is an infection caused by the dengue virus (DENV), an enveloped virus belonging to the family *Flaviviridae*. It is primarily transmitted by *Aedes aegypti* and, to a lesser extent, *Aedes albopictus* mosquitoes. DENV contains a single-stranded RNA genome with positive polarity of approximately 10.6 kb, encoding three structural proteins (capsid, pre-membrane/membrane and envelope) and seven non-structural proteins, distinguishing four viral serotypes: DENV-1, DENV-2, DENV-3, and DENV-4 [1].

Vector-borne diseases such as dengue involve complex bipartite transmission networks between humans and vectors that can be modeled to elucidate disease spread. Because DENV transmission is driven by both human-to-human interactions and human-vector contact, and is strongly influenced by population density and environmental factors, effective vector control is crucial for outbreak mitigation [2,3].

Reflecting global trends, dengue has surged in Latin America and is now the fastest-spreading vector-borne disease in the region. Its global incidence has increased 30-fold, driven by the spread of *Aedes aegypti* mosquitoes and the emergence of multiple DENV serotypes and genotypes, reaching 50–100 million cases in 2024, with over 90% occurring in the Americas. Several countries in the region have declared states of emergency in response. Data from the World Health Organization (WHO) from January to November 2024 reported 13.8 million cases and 9990 deaths globally [4,5]. The Americas accounted for 12.7 million cases and 7713 deaths, with Brazil reporting a record-breaking 6 million probable cases and over 5000 deaths, followed by Argentina, Peru, Colombia, Paraguay, and Ecuador [6].

All four DENV serotypes now co-circulate in the region, heightening the risk of severe illness [5,6]. The sustained spread of dengue is fueled by ecological, climatic and socioeconomic conditions (such as poor housing, inadequate sanitation, and limited access to healthcare) that support vector proliferation and hinder outbreak response [4,6].

In 2023, the Pan American Health Organization (PAHO) and the WHO classified dengue in the Americas as a grade 3 emergency, the highest level under the WHO’s Emergency Response Framework, indicating a need for major coordinated response from regional offices. As a result, PAHO has urged countries to intensify mosquito control efforts, and strengthen surveillance, early diagnosis, and timely treatment [4,5].

Notably, whereas the 2018 PAHO-integrated arbovirus strategy included vaccination as a component of dengue prevention, the updated 2020 strategy excludes vaccination as an intervention [7,8]. This shift is not aligned with current dengue control recommendations and the evolving regional epidemiological landscape. The global strategy for dengue prevention and control, as outlined by WHO (2012–2020), is multifaceted and integrated, combining vector control via integrated vector management (IVM), vaccination (as indicated for eligible populations), robust surveillance, and sustained community engagement—underscoring the importance of these pillars working cohesively [2,9].

Given the complex and uncontrolled nature of the dengue outbreak in Latin America, innovative strategies are essential to strengthening public health interventions and limiting dengue transmission. This narrative review synthesizes expert guidance and recommendations from the Dengue Task Force into a multisectoral, implementation-oriented framework spanning surveillance, vector control, clinical management, vaccination, environmental management, and governance, and discusses how that framework can be adapted to the region’s varied epidemiological, ecological, and social contexts.

## 2. Methods and Expert Deliberation

In March 2025, the Americas Health Foundation (AHF) convened the Latin America Dengue Task Force in Bogotá, Colombia, bringing together a multidisciplinary group of regional experts in clinical care, epidemiology, entomology, surveillance, vaccination, public health, and policy from multiple Latin American countries. The meeting aimed to identify major barriers to dengue prevention and control and develop practical recommendations for the region.

To inform the discussions, AHF conducted a targeted narrative literature review on dengue epidemiology, surveillance, vector control, vaccination, diagnostics, health systems, and environmental and social determinants. Searches drew on databases such as PubMed and Scopus, and on institutional sources including the WHO, PAHO, and national Ministries of Health. This is a narrative, non-systematic review; no registered protocol or formal systematic review methods were used.

Barriers and recommendations were developed through small-group thematic sessions, followed by plenary discussion and iterative revision after the meeting through email exchanges among Task Force members. Broad agreement was reached through discussion and revision, without formal voting or Delphi-style consensus procedures. This approach has limitations, including possible selection bias in expert participation and the inherent constraints of a narrative rather than systematic review design.

## 3. Key Barriers and Challenges

### 3.1. Surveillance and Information System Gaps

Robust surveillance systems are essential for effective dengue prevention and control. However, important challenges persist across Latin America, including underreporting, delays in case notification, fragmented information systems, limited integration of entomological and climate data, and persistent territorial inequities. These gaps constrain timely decision making and weaken the ability of health systems to anticipate and respond to outbreaks. Table 1 summarizes key gaps and barriers to integrated dengue control in Latin America. 

#### 3.1.1. Misdiagnosis, Underreporting and Passive Surveillance

Most DENV infections are asymptomatic or mildly symptomatic, leading to extensive underreporting. Cohort studies estimate that 82% of primary and 59% of secondary infections are asymptomatic; cluster studies report 78% and 43%, respectively. Since more than 80% of infections occur in individuals with mild or no symptoms who rarely seek medical care, official statistics capture only a fraction of actual cases, distorting risk assessments [10]. Cohort-based analyses comparing active surveillance with national notification systems suggest that actual dengue incidence in Latin American countries may be several-fold higher than reported, with estimated expansion factors ranging from single-digit to >20 in settings such as Brazil, Colombia and Nicaragua [11]. In many countries, dengue is a legally notifiable disease, but weak enforcement mechanisms and insufficient regulatory oversight contribute to widespread underreporting. Strengthening legal frameworks to mandate standardized reporting, ensure adequate monitoring and provide appropriate incentives is critical for effective surveillance.

Dengue’s non-specific clinical presentation overlaps with other febrile illnesses (such as Zika, chikungunya, Oropouche, Mayaro, COVID-19, malaria, typhoid, leptospirosis, influenza, and even autoimmune conditions like systemic lupus erythematosus), causing frequent misdiagnosis. Up to 25% of cases may be misclassified based solely on clinical assessment, leading to unnecessary hospitalizations, higher costs, and complications such as fluid overload [12,13].

Advanced diagnostics are often limited to regions with laboratory infrastructure. In most resource-constrained settings, clinicians rely on syndromic surveillance and judgment, with wide diagnostic uncertainty. Standardized reporting systems, harmonized case definitions, and improved diagnostic access are crucial for accurate burden estimation, timely outbreak detection, and coordinated interventions. Inconsistencies obscure the true incidence and undermine regional comparability.

In much of Latin America, dengue surveillance is primarily passive, capturing cases only when individuals seek care and undergo clinical or laboratory confirmation [14]. Though cost-effective, this system fails to detect community-level transmission and introduces delays in data reporting. Active surveillance (community-based screening and testing) is rarely used, despite its capacity to generate more accurate epidemiological insights.

Underreporting in passive national surveillance systems arises from organizational, cultural, patient-related, and legal factors (Table 2). Detection rates vary with context: during outbreaks, heightened awareness improves reporting, whereas endemic periods encourage complacency [15,16]. Conversely, epidemics can also trigger over-reporting due to increased diagnostic suspicion [17]. In Brazil, Colombia, and Mexico, actual case numbers are estimated to be 10–25 times higher than official reports, underscoring the need for more robust, proactive surveillance strategies [18]. Because IVM relies on robust epidemiological and entomological surveillance to guide interventions, these weaknesses directly undermine evidence-based vector control.

#### 3.1.2. Timeliness of Reporting and Data Quality

Timely access to epidemiological data is vital for effective outbreak response, yet reporting lags are common. Cases may take weeks to be processed and reported, particularly in rural or resource-limited settings where manual or paper-based reporting is still in use. These delays weaken early warning systems, making it difficult to predict epidemics or deploy resources efficiently [17,19,20,21].

Historical outbreaks in the region have shown that case surges may occur before public health alerts are issued [22]. Furthermore, most passive systems focus on severe cases, biasing serotype data and delaying the detection of new viral strains, as documented in published analyses of regional dengue resurgence events [23]. Standardized reporting systems, harmonized case definitions, and improved diagnostic access are therefore crucial for accurate burden estimation, timely outbreak detection, and coordinated interventions, as inconsistencies obscure true incidence and undermine regional comparability.

#### 3.1.3. Entomological and Climate-Informed Surveillance

There is often poor alignment between epidemiological surveillance and entomological monitoring, limiting the ability to forecast hotspots accurately. Dengue control campaigns may therefore direct insecticide spraying to low-risk areas while missing true transmission zones. Moreover, the timing and frequency of insecticide applications may not coincide with peak vector activity, further undermining control measures. Misalignment of intervention timing with mosquito activity reduces control effectiveness and wastes resources [24]. Entomological monitoring, crucial for assessing *Aedes aegypti* resistance and breeding sites, is often inconsistent due to funding shortages [25,26,27]. At the same time, climate change and urbanization increasingly complicate vector dynamics, though integration of climate adaptation strategies into surveillance programs remains limited [9]. Strengthening response agility will require standardized entomological monitoring, incorporation of climate indicators, and interoperable platforms that bring these data together in real time.

#### 3.1.4. Equity and Territorial Data Gaps

Data collection commonly prioritizes urban areas, leaving rural and marginalized populations underrepresented. This urban bias skews public health strategies and widens health disparities, as vulnerable rural communities (often with inadequate housing, poor healthcare access, and socioeconomic disadvantages) receive delayed or insufficient interventions [28,29]. Ensuring equitable data collection and integrating local community knowledge into public health planning are essential to reduce inequities and improve responsiveness across diverse populations [22].

#### 3.1.5. Fragmented Information Systems and Data-Driven Decision Making

Effective dengue surveillance relies on strong coordination between laboratories, that confirm cases, and clinical services, that diagnose and report them. Weak linkages between these actors delay case confirmation and, in turn, outbreak recognition. When laboratories fail to provide timely feedback, clinicians lose confidence in the system and may stop reporting suspected cases, undermining surveillance accuracy. These delays also have operational consequences: vector control measures and resource allocation, such as insecticide distribution and medical supplies, are often misdirected in the absence of real-time data on confirmed cases [30].

Fragmentation across the wider data ecosystem compounds these challenges. Ministries, municipalities, and research groups often work in silos, slowing information sharing and reducing the effectiveness of outbreak coordination. These gaps risk not only late detection but also public confusion, as inconsistent case reporting weakens trust and lowers compliance with prevention guidance [25,30]. In an increasingly interconnected region, the lack of mechanisms for cross-border data sharing further limits timely response, as dengue transmission frequently extends beyond national boundaries. Strengthening regional cooperation in digital health and enabling secure, standardized exchange of health data can improve outbreak preparedness, support routine surveillance, and enhance coordinated responses across countries [31].

Digital innovations offer pathways to overcome these barriers. Tools like geospatial information systems (GIS) and artificial intelligence (AI) could support predictive modeling for more proactive vector control, yet their adoption remains limited [32]. Strengthening response agility will require investment in interoperable data platforms, decentralized diagnostics, and standardized entomological monitoring to ensure that information is timely, actionable, and shared across stakeholders [33].

Many countries still confront fragmented information systems characterized by under-reporting and delayed outbreak responses, limiting their capacity to use surveillance information to guide policy and operational decisions.

### 3.2. Limited Laboratory Capacity

Diagnosis remains constrained by limited laboratory infrastructure. Affordable, reliable diagnostic tools suitable for primary care are urgently needed to support both patient management and surveillance. Access to PCR, ELISA, and other advanced assays is typically centralized, which delays confirmation and complicates outbreak detection. Weak coordination between public health labs and clinical facilities results in fragmented reporting, while the use of different data formats hampers integration [9,34,35,36]. Manual data transfer further slows responses and increases errors.

### 3.3. Insufficient Entomological Surveillance

Monitoring of *Aedes aegypti* populations is inconsistent across the Americas. Traditional indicators such as the Breteau and House indices are collected irregularly and often with varying methodologies, limiting their comparability and predictive utility [37,38,39]. The lack of real-time geospatial mapping hinders targeted interventions and reduces the ability to prioritize high-risk areas or assess control impact.

### 3.4. Vector Control Obstacles

Efforts to control dengue vectors face mounting obstacles that undermine both traditional and novel interventions. The most pressing biological challenge is widespread insecticide resistance: studies report resistance to permethrin in over 80% of *Aedes aegypti* populations in Brazil, Colombia, and Mexico, with particularly dramatic increases documented in Puerto Rico between 2012 and 2022 [25,26,27]. This trend severely limits the effectiveness of cornerstone chemical control strategies.

Beyond resistance, traditional vector control approaches often rely on top–down interventions that marginalize community participation. Rapid and often unplanned urbanization further reduces effectiveness, as inadequate water storage, poor waste management, and dense settlement patterns create abundant mosquito-breeding sites that are difficult to eliminate through routine spraying or larval control campaigns [40,41].

In response, new biological and technological tools have been developed, but these innovations present their own challenges. Biological control of dengue through the release of *Aedes aegypti* mosquitoes infected with the bacterium *Wolbachia* represents the only currently available control strategy capable of providing sustained, long-term impact [42,43,44,45]. While implementation requires significant upfront investment, logistical coordination, sustained community acceptance, and clear regulatory pathways, once released, Wolbachia is vertically transmitted to subsequent mosquito generations, enabling the intervention to remain effective for many years by inhibiting viral replication within the vector [46]. This effect extends beyond DENV to Zika, chikungunya, and yellow fever viruses. Studies conducted in Asia, Australia, and the Americas have demonstrated reductions in dengue incidence exceeding 90% following the release of Wolbachia-infected mosquitoes [47]. Cost-benefit analyses indicate that the initial investment required for implementation is recovered within approximately 20 months, followed by a sustained positive annual return that persists for more than ten years [11].

Similarly, approaches such as Attractive Toxic Sugar Baits (ATSBs) and novel trapping devices show promise but face issues of cost-effectiveness, scalability in diverse environments, and risks to non-target insect populations [44]. Their deployment also depends on robust local capacity for placement, maintenance, and monitoring [44,48].

Finally, environmental management strategies, though crucial for sustainable dengue control, confront long-standing political, financial, and social obstacles. Enforcing building codes, upgrading water and waste infrastructure, and extending services to informal settlements require substantial investment and governance capacity [41]. Moreover, sustaining behavior change at the community level remains a persistent difficulty, particularly in marginalized settings where structural constraints limit choices.

Taken together, these barriers highlight the need for integrated, approaches that combine technical innovation with systemic infrastructural improvements and genuine community engagement. Without tackling both biological resistance and socio-environmental determinants, vector control alone cannot provide a durable solution to dengue transmission.

### 3.5. Vaccines and Treatment Challenges

Dengue vaccination is a cornerstone of integrated dengue control strategies, endorsed for its potential to substantially reduce disease burden. However, vaccines differ in their safety profiles, efficacy, and recommended use, requiring clear communication to avoid misconceptions. Current vaccines available include the first generation Dengvaxia, the next-generation Qdenga^®^, and the Brazilian-developed and produced vaccine Butantan-DV (Table 3).

While dengue vaccines mark a significant advance, challenges remain. Vaccine hesitancy, cost, supply logistics, and the need for robust pharmacovigilance systems must be addressed to maximize impact. Critically, vaccination must be integrated into comprehensive dengue prevention frameworks that include vector control, surveillance, health system strengthening, and community engagement. In this context, establishing clear regulatory pathways for vaccine authorization, indication, and monitoring is essential, not only to ensure safety and efficacy but also to reinforce transparency and rebuild public trust. Strengthening legal frameworks around dengue immunization can help address existing concerns, particularly in countries where past experiences have weakened confidence in vaccination programs.

### 3.6. Climate and Environmental Factors

Dengue transmission dynamics and control efforts are increasingly challenged by a range of external environmental and societal disruptions (such as climate variability, extreme weather events, and global health emergencies) that alter vector ecology, hinder control measures, and strain public health systems.

Rising temperatures and shifting rainfall patterns are amplifying the ecological suitability for *Aedes aegypti* and, by extension, dengue transmission. Temperature influences both mosquito survival and viral dynamics: within the optimal range of 20 °C to 26 °C, mosquito longevity and biting rates increase, and the extrinsic incubation period shortens, facilitating more rapid transmission. Temperatures outside this range, however, can reduce survival and transmission efficiency.

Rainfall and humidity also play critical roles. Adequate rainfall sustains larval habitats, while higher relative humidity prevents adult mosquitoes from desiccating. In contrast, extremely dry conditions can shorten mosquito lifespan by accelerating fluid loss through the spiracles, which lack effective regulatory mechanisms [56]. These climate–mosquito interactions underscore the importance of considering local environmental contexts. In tropical regions where temperature fluctuations are modest, mean temperature may have less impact than rainfall variability, humidity, and microhabitat availability [57]. Further research is needed to disentangle these interacting environmental and entomological factors and quantify their specific relationships with dengue transmission.

In addition to gradual climate shifts, extreme weather events pose significant threats to dengue control. Hurricanes, heavy floods, and other disasters disrupt ongoing vector control efforts, displace populations, and create extensive new breeding sites through stagnant water. Such post-disaster conditions have repeatedly triggered localized dengue surges that overwhelm already strained public health systems [58].

Together, these findings highlight climate variability and extreme events as critical drivers that complicate vector management and disease prediction, reinforcing the need to integrate climate-informed strategies into dengue surveillance and response.

#### Pandemic Disruptions to Dengue Control

The COVID-19 pandemic further exemplifies how external crises can reshape dengue transmission dynamics. Lockdowns and mobility restrictions altered patterns of human–mosquito contact, sometimes reducing exposure but also disrupting routine vector surveillance and control operations. Movement restrictions, diversion of resources to the COVID-19 response, and reduced community engagement weakened mosquito control efforts and likely increased breeding opportunities [59].

Health system disruptions also affected dengue surveillance and clinical management. Case detection and reporting were delayed or underreported in several regions, obscuring early warning signals for outbreaks [59]. Overlapping clinical features between COVID-19 and dengue added diagnostic complexity, further straining already overstretched healthcare systems [60]. At the policy level, attention and funding often shifted toward pandemic response, temporarily diminishing focus on arboviral disease programs [59].

These pandemic-induced disruptions highlight the vulnerability of dengue control to global health emergencies. Strengthening resilience through integrated, flexible surveillance systems and adaptable vector control programs is critical to safeguarding against both environmental shocks and future pandemic pressures.

### 3.7. Political and Financial Barriers

Dengue control often suffers from cyclical attention, with political engagement surging during outbreaks but waning in inter-epidemic periods. This reactive approach undermines sustainable prevention efforts, such as vector control and community education. Competing public health priorities (including HIV/AIDS, malaria, and emerging zoonotic diseases) often divert policy focus and funding away from dengue [11]. Without strong high-level political endorsement, dengue programs struggle to secure the legislative and administrative support necessary for sustained interventions [11].

Effective dengue control requires coordinated action across multiple government sectors, including health, environment, urban planning, and education. However, overlapping jurisdictions and bureaucratic inefficiencies often lead to fragmented efforts [61]. Health ministries may prioritize insecticide spraying, while municipal authorities lack the capacity or mandate to enforce sanitation regulations that reduce mosquito-breeding sites. Frequent leadership changes and weak enforcement of public health regulations exacerbate these challenges.

Guidelines for mosquito-proofing water storage containers are often disregarded due to lax oversight, and penalties for maintaining mosquito-breeding sites in residential or commercial areas are rarely enforced, further diminishing compliance with public health regulations.

Dengue control programs face chronic budget constraints, with funding often tied to donor priorities rather than local epidemiological needs [33]. Many low- and middle-resourced countries rely on external funding, which is often inconsistent and earmarked for short-term emergency responses rather than long-term prevention [34]. Domestic health budgets in endemic areas often fall short of covering comprehensive vector control, diagnostics, and public awareness campaigns [11].

Financial resources, when available, are sometimes inefficiently allocated, favoring costly reactive interventions such as fumigation during outbreaks over more cost-effective preventive strategies like community-based larval source reduction [62]. Additionally, hospital-based dengue management commands disproportionate funding compared to IVM, despite evidence supporting IVM’s effectiveness in reducing long-term transmission [63].

Socioeconomic disparities further exacerbate the burden of dengue. Marginalized communities often lack access to preventive healthcare and reside in environments conducive to mosquito breeding. Government spending on dengue control often favors urban centers, leaving rural and peri-urban populations underserved [64,65]. Furthermore, out-of-pocket healthcare expenses for dengue treatment can push low-income households into poverty, perpetuating a vicious cycle of vulnerability.

Addressing institutional fragmentation requires not only political will and intersectoral collaboration, but also the establishment of clear legal and governance frameworks. Defining the roles and responsibilities of national, subnational, and local authorities (as well as across health, environment, housing, and sanitation sectors) can strengthen coordination and accountability. Regulatory clarity is essential to operationalize a sustainable and multisectoral dengue control strategy.

This challenge also highlights the need for stronger regional coordination across Latin America. Regional coordination is essential for effective dengue control because dengue transmission, population mobility, vector spread, and surveillance challenges frequently cross national borders. Other regions have established institutional mechanisms to support cross-border surveillance, technical cooperation, and coordinated outbreak response [6,66,67,68] whereas Latin America still lacks a comparably strong and clearly mandated regional framework for epidemic coordination. Strengthening regional collaboration, interoperable surveillance systems, and shared response strategies would help countries detect outbreaks earlier, align public health actions, and improve the overall effectiveness of dengue prevention and control efforts.

### 3.8. Socioeconomic and Behavioral Factors

Socioeconomic and behavioral factors are closely intertwined and play a critical role in shaping the burden of dengue. Poor communities, often lacking adequate sanitation and healthcare resources, are more vulnerable to dengue transmission. Human behaviors, such as water storage practices and the presence of potential mosquito-breeding sites, can significantly influence local mosquito populations and drive transmission dynamics. Limited access to quality healthcare and diagnostic services further delays treatment, allowing infected individuals to serve as reservoirs for ongoing spread [65,69].

Poverty, low income, limited education, and rapid urbanization contribute to inadequate housing, poor sanitation, and restricted healthcare access, creating environments conducive to mosquito breeding and elevated dengue risk. The widespread use of open water containers, water storage management, and gaps in knowledge about practices are major contributors to transmission [70]. Additionally, higher density and household crowding can increase the risk of exposure to infected mosquitoes [71,72].

Insufficient community knowledge about dengue transmission and prevention can foster behaviors that increase vulnerability to mosquito bites and infection. Low engagement in vector control measures, such as the use of mosquito nets and the elimination of breeding sites, undermines efforts to reduce mosquito populations. Increased global travel and trade also facilitate the spread of DENV to new regions, particularly where preventive strategies are lacking [73].

These intertwined factors create complex barriers that require multidisciplinary approaches addressing both structural inequalities and health literacy. Targeted interventions, community education, and improved infrastructure are essential to reducing dengue transmission and improving public health outcomes.

In addition to social and environmental factors, legal determinants of health (such as the right to adequate housing, access to safe water, and basic sanitation) are essential components of dengue prevention. Recognizing these as State obligations under frameworks like General Comment No. 14 of the United Nations Covenant on Economic, Social and Cultural Rights reinforces the need for regulatory action to address structural drivers of vulnerability [74].

### 3.9. Systemic and Cross-Cutting Barriers

#### 3.9.1. Healthcare System Weaknesses

Diagnostic delays, limited workforce capacity, and weak health information systems undermine timely detection and management. Outbreaks regularly overwhelm fragile health systems, highlighting overreliance on reactive, hospital-based care rather than proactive prevention [75].

Furthermore, the coordination between IVM and primary healthcare remains inadequate, perpetuating a reliance on reactive rather than preventive measures [76]. Without sustained investment in surveillance, workforce capacity, and community-based vector control, dengue-endemic health systems will continue to face cyclical crises, exacerbating morbidity and mortality in vulnerable populations [76].

#### 3.9.2. Mobility-Driven Transmission

Population mobility significantly influences dengue spread. For example, increased migration over the last decade from endemic South American countries with documented co-circulation of all four DENV serotypes (such as Brazil, Venezuela, and Colombia) may have contributed to the introduction of DENV-3 into neighboring countries and intensified outbreaks, with hospitalization rates reported to be three times higher than those from endemic transmission [77]. Similarly, seasonal labor movements create persistent transmission corridors, while even routine house-to-house mobility drives localized spread, patterns poorly captured by traditional surveillance [71].

#### 3.9.3. Fragmented Multisectoral Coordination

IVM, promoted by the WHO, is the recommended strategy for dengue control, emphasizing evidence-based decision-making, intersectoral collaboration, and community engagement [78,79]. However, its implementation in Latin America has been hindered by fragmented multisectoral coordination, inconsistent political commitment, and systemic health system weaknesses [64].

Effective IVM requires strong collaboration between health, environment, urban planning, education, and community stakeholders. However, in Latin America, dengue control efforts are often siloed within Health Ministries, with limited engagement from other sectors. Municipal governments, which are critical for localized vector control, frequently lack the technical and financial capacity to sustain IVM. Additionally, political cycles disrupt long-term planning, as dengue prevention is often deprioritized outside of epidemic periods.

While national dengue programs exist, decentralization has led to uneven implementation, with poorer municipalities struggling to maintain surveillance and vector control. Similarly, in Central America, vertical top–down approaches have weakened local adaptability, reducing community trust and participation [2]. Without institutionalized intersectoral mechanisms, IVM remains reactive rather than preventive.

Importantly, fragmented multisectoral coordination not only impacts IVM but undermines the entire spectrum of integrated dengue management, including diagnosis, clinical care, and vaccination efforts. This fragmentation contributes to siloed programs, inconsistencies in clinical management, gaps in surveillance, disparities in vaccination strategies, and limited uptake of new countermeasures [80]. These systemic gaps are highlighted by contrasts between frameworks: while IMS-Arbovirus focuses narrowly and omits vaccination [8], previous IMS-Dengue and the WHO Global Strategic Plan for Dengue and Aedes Response 2024 explicitly call for multisectoral coordination across all interventions (including vaccines, clinical management, surveillance, and vector control) recognizing that effective dengue control depends on harmonized action across all sectors [59].

WHO’s Strategic Preparedness, Response and Resilience Plan for dengue and other *Aedes*-borne arboviruses comprises five key components essential for a successful outbreak response:

1. **Emergency coordination**: Establishing leadership and coordination activities.
2. **Collaborative surveillance**: Developing and using tools for early detection and control of dengue and other *Aedes*-borne outbreaks, including strengthened indicator and event-based surveillance, epidemiological analysis, laboratory diagnostics, and field investigations.
3. **Community protection**: Engaging communities through active dialogue and local adaptation of prevention and response measures, including mosquito population control.
4. **Safe and scalable care**: Ensuring effective clinical management and resilient health services to ensure patients can receive adequate care and prevent illness and death.
5. **Access to countermeasures**: Promoting research and innovation for improved treatments and effective vaccines against these diseases.

Building robust multisectoral coordination mechanisms that span vector control, clinical management, diagnosis, and vaccination is therefore essential for comprehensive and sustainable dengue prevention and control in the region.

### 3.10. Community Engagement

Community participation is crucial not only for IVM but also for the success of broader preventive measures and public health awareness campaigns related to dengue control. However, many programs fail to sustain engagement beyond short-term campaigns [40]. Cultural perceptions of dengue risk vary widely: some populations normalize the disease, while others harbor distrust toward government interventions [69]. Moreover, household-level prevention behaviors, such as water storage practices, sometimes conflict with official vector control recommendations. Without culturally adapted communication and participatory approaches, sustained behavioral change remains limited.

These intersecting barriers underscore the need for an integrated, regionally coordinated response, which the following recommendations from the AHF Stop Dengue Task Force aim to support.

## 4. Recommendations by Barrier Domain

**Figure 1:**
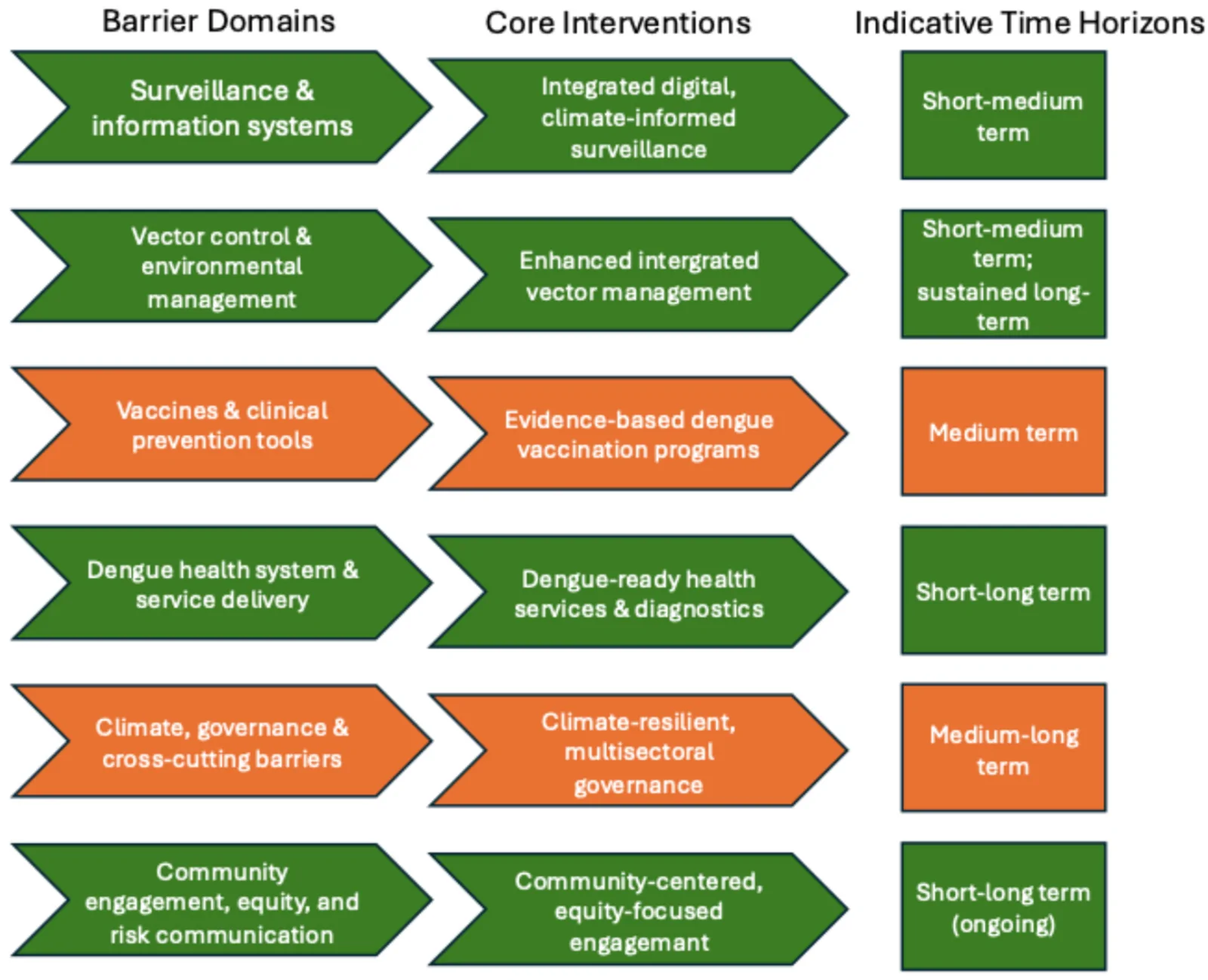
Implementation roadmap linking major dengue control domains in Latin America (**left**) to core intervention packages (**center**) and indicative implementation horizons (**right**).

Addressing the complex challenges of integrated dengue control in Latin America requires a strategic, multifaceted roadmap. The recommendations below are organized to mirror the major barrier domains described in Section 2, moving from surveillance and vector control through vaccination, health systems, governance, and equity. This approach must prioritize strengthening surveillance, deploying innovative vector control technologies, addressing social and environmental determinants, and enhancing regional coordination. The following expert recommendations from AHF’s Stop Dengue Task Force provide a focused framework and timing to advance dengue prevention and control across the region (Figure 1).

### 4.1. Surveillance and Information System Gaps

Building directly on the surveillance and information system gaps outlined in Section 3.1, these recommendations aim to strengthen collaborative, real-time, and interoperable dengue surveillance across the region. Robust, real-time surveillance is essential for early outbreak detection and data-driven decision-making. Sustaining these systems over time will require long-term funding commitments, interoperable data standards, and strong political will to ensure coordinated, continuous functionality across the region. Strengthening surveillance also requires moving beyond defining which indicators should be measured to clarifying the outcomes that surveillance is intended to inform. Surveillance systems should therefore integrate both population-level and territorial dimensions of transmission and be designed to support timely, action-oriented public health responses. Drawing on WHO and PAHO surveillance frameworks [7], the Task Force endorses a structured, multidomain monitoring approach to guide integrated dengue surveillance across the region (Table 4).

To improve regional monitoring and response, the following actions are recommended:

- **Invest in digital surveillance platforms:** Implement tools like Brazil’s Infodengue and Argentina’s Sistema Nacional de Vigilancia de la Salud to integrate clinical, laboratory, and entomological data, reducing reporting delays by 30–50%.
- **Leverage mobile health tools:** Enable community-based reporting through smartphones to expand case detection in remote or underserved areas.
- **Apply machine learning and AI:** Integrate predictive models that analyze historical case data, climate patterns, and social determinants to anticipate outbreaks and trigger early interventions. Their adoption should be accompanied by robust data governance frameworks to ensure confidentiality, responsible interoperability, and compliance with regional personal data protection regulations.
- **Enable cross-sectoral data sharing:** Strengthen collaboration between health, environmental, and municipal agencies to ensure comprehensive surveillance.
- **Establish early-detection networks:** Monitor high-transmission settings (such as schools and workplaces) to rapidly identify and report cases.
- **Expand access to genomic sequencing:** Track dengue serotype distribution and monitor for the emergence of new strains, to enhance outbreak preparedness.

### 4.2. Vector Control and Environmental Management

An IVM approach is essential for sustainable dengue control. Combining biological, environmental, technological, and community-based interventions can significantly reduce mosquito populations and transmission.

#### 4.2.1. Biological Tools

In response to the entomological weaknesses, vector control obstacles, and environmental determinants highlighted in Section 3.1.3, Section 3.3 and Section 3.5, this section focuses on reinforcing integrated vector management and environmental interventions. Biological strategies such as Wolbachia-infected mosquito releases offer an important opportunity to achieve sustained reductions in dengue transmission.

The following actions are recommended:

- **Expand biological control tools:** Scale up *Wolbachia*-infected mosquito releases, proven effective in cities like Medellín and Rio de Janeiro.
- **Scale up biological control methods:** Expand the use of Wolbachia-infected mosquitoes, as implemented by the World Mosquito Program in Brazil and Colombia, to sustainably reduce *Aedes aegypti’s* ability to transmit dengue [64].
- **Deploy technology-enabled interventions:** Use drones for targeted larvicide application and AI-powered traps to monitor and suppress mosquito populations.
- **Support community and infrastructure improvements:** Pair household-level education and clean-up efforts with upgrades to water storage, drainage, and waste management to eliminate mosquito-breeding sites sustainably.

#### 4.2.2. Novel Technologies

Novel tools such as ATSBs and trapping devices have emerged as promising technologies to complement traditional dengue vector control [39]. Effective use of ATSBs and traps depends on robust local capacity for correct placement, maintenance, and monitoring, and on community acceptance, which can be difficult to sustain in under-resourced settings. Consequently, these technologies should be deployed as part of integrated, locally adapted vector management programmes with explicit evaluation of epidemiological outcomes, rather than as stand-alone solutions.

Recommended actions include:

- **Deploy ATSBs:** Utilize low-cost, eco-friendly traps to target adult mosquitoes in urban and peri-urban environments.

#### 4.2.3. Environmental Management and Community Engagement

Environmental management remains a foundational component of dengue prevention, particularly in settings where inadequate water storage, poor drainage, and waste accumulation sustain mosquito breeding.

Recommended actions include:

- **Adopt AI-powered surveillance tools:** Implement smart traps such as Gravitraps linked to cloud dashboards to track and suppress mosquito populations using real-time data.
- **Improve environmental and structural conditions:** Enforce building codes for mosquito-proof water storage and promote better waste and water management practices to eliminate breeding sites. Drones may be used to apply larvicides in hard-to-reach urban areas to improve coverage, efficiency, and safety.
- **Mobilize communities through technology:** Introduce gamified mobile apps that reward users for identifying and eliminating breeding grounds, increasing public engagement and accountability.
- **Sustain behavior change through education:** Launch community campaigns that build awareness and empower residents to maintain long-term preventive practices.

Key innovative and integrated vector control strategies discussed by the expert panel are summarized in Table 5.

### 4.3. Vaccines and Clinical Prevention Tools

Addressing the vaccine and treatment challenges described in Section 3.5, these recommendations seek to optimize the use of dengue vaccines and complementary prevention tools in Latin America. The introduction of newer dengue vaccines offers a significant opportunity for prevention, but success depends on strategic deployment and public trust.

Recommended actions include:

- **Develop national vaccination guidelines:** Prioritize high-transmission areas while ensuring risk–benefit considerations guide implementation.
- **Implement safety monitoring:** Establish robust pharmacovigilance systems to track and communicate vaccine safety data, addressing public concerns in real time.
- **Ensure equitable access:** Use school-based campaigns and mobile units to reach remote or underserved populations, minimizing geographic and financial barriers.
- **Build vaccine confidence:** Engage communities through tailored messaging and trusted messengers to address vaccine hesitancy, especially in marginalized populations.

### 4.4. Strengthening Dengue Health System and Service Delivery

In light of the health-system weaknesses, diagnostic gaps, and service-delivery constraints discussed in Section 3.1.1 and Section 3.9, this section proposes actions to strengthen dengue care and service delivery. Capacity-building for healthcare workers is essential to improve dengue diagnosis, particularly at the primary care level, where febrile illnesses like dengue, Zika, and chikungunya are first evaluated. Regional collaboration platforms can support the standardization of diagnostic practices, facilitate knowledge exchange, and improve data sharing across borders.

#### 4.4.1. Capacity Building and Regional Collaboration

Key strategies include:

- **Implement standardized case definitions:** Promote consistent diagnostic criteria for dengue, Zika, Oropuche, and chikungunya to reduce misclassification and improve reporting accuracy.
- **Expand access to virtual training platforms:** Leverage tools such as PAHO’s Virtual Campus to provide ongoing education to frontline health workers, especially in underserved or rural areas.
- **Enable telemedicine consultations:** Support remote clinical decision-making by enabling virtual consultations for complex or ambiguous cases.
- **Foster joint surveillance initiatives:** Encourage regional partnerships to monitor dengue serotype distribution, detect outbreaks early, and coordinate public health responses.

#### 4.4.2. Prioritization of Febrile Syndromes

Febrile illnesses in vulnerable groups such as children, pregnant women, the elderly, and immunocompromised individuals are often underreported due to misdiagnosis or limited access to care. Prioritizing these populations in dengue strategies can reduce the risk of severe outcomes. Recommended actions include:

- **Strengthen febrile illness protocols in primary care:** Implement standardized case management pathways based on the WHO dengue guidelines (2009/2021), including risk-stratification tools to identify severe cases early.
- **Expand access to rapid diagnostic tests:** Make NS1 antigen testing available at the point-of-care to support quicker diagnosis and timely intervention.
- **Implement community-based fever surveillance:** Establish fever monitoring systems in high-risk areas to facilitate early detection and response before outbreaks escalate.

#### 4.4.3. Improving and Decentralizing Laboratory Diagnostics

Accurate and timely laboratory diagnostics are central to effective dengue control and outbreak management. Expanding and decentralizing diagnostic capacity can reduce delays and improve responsiveness, especially in remote or underserved regions. Key strategies include:

- **Scale up access to NS1 antigen and PCR testing**: Equip regional laboratories with tools for early detection and serotype identification to improve case confirmation and surveillance [64].
- **Develop decentralized diagnostic networks:** Reduce turnaround times by enabling local testing, which minimizes dependence on centralized labs.
- **Deploy mobile and point-of-care testing (POCT) units:** Introduce validated POCT tools suited for low-resource settings to expand access in outbreak-prone areas.
- **Invest in laboratory infrastructure and training:** Strengthen diagnostic capacity by upgrading facilities and providing technical training to local health personnel.

#### 4.4.4. Updating Clinical Protocols

Dengue’s clinical presentation varies by serotype and host factors, requiring protocols that reflect current epidemiological trends and population vulnerabilities. To improve patient outcomes and reduce mortality, especially in high-risk groups, the following actions are recommended:

- **Revise protocols to reflect circulating serotypes:** Update guidelines to account for the rising prevalence of DENV-3 and DENV-4 in Central America, which are associated with atypical presentations such as post-febrile thrombocytopenia.
- **Adapt care for high-risk populations:** Tailor clinical guidance for children, pregnant women, people with comorbidities, and older adults, who are at greater risk of severe outcomes and death.
- **Integrate warning signs and risk tools:** Incorporate new clinical indicators and stratification models into national protocols to support early identification of severe cases.
- **Embed protocols into health systems:** Link updated guidelines to electronic health records (EHRs) and clinical decision-support tools to standardize care across providers and facilities.
- **Promote continuous training:** Ensure frontline providers receive regular updates on evolving clinical presentations and treatment recommendations.

### 4.5. Climate, Governance, and Cross-Cutting Implementation Barriers

To respond to the climate-related risks, political and financial barriers, and other cross-cutting obstacles summarized in Section 3.1, Section 3.6, Section 3.7 and Section 3.9, these recommendations emphasize governance reforms, climate-informed planning, and multisectoral coordination. Dengue transmission is deeply influenced by climate variability and socioeconomic conditions. Structural conditions, such as inadequate housing, poor access to water and sanitation, and climate vulnerability, must be acknowledged and addressed in public health planning. Embedding these determinants into policy frameworks can help create more proactive, equitable dengue control strategies.

#### 4.5.1. Models and Policy

Key recommendations include:

- **Integrate climate data into forecasting models:** Use predictive tools that incorporate climate indicators (e.g., El Niño–Southern Oscillation patterns) alongside epidemiological data to improve early warning systems and regional outbreak preparedness.
- **Develop Dengue Vulnerability Indices (DVIs):** Combine data on exposure, susceptibility, and adaptive capacity to generate composite risk scores. DVIs can help identify high-risk areas and guide targeted interventions such as vector control, public health education, and resource distribution.
- **Promote intersectoral policy coordination:** Link public health with urban planning, water and sanitation, and climate resilience to reduce environmental risk factors and improve community-level preparedness. This approach should support risk- and data-driven proactive management, moving away from reactive “waiting periods” between transmission seasons toward continuous strengthening of system resilience and preparedness.
- **Leverage AI and big data for early detection:** Emerging technologies can improve epidemic prediction and generate faster, more accurate alerts. However, their effectiveness depends on addressing challenges such as fragmented data systems, infrastructure limitations, and ethical concerns.
- **Ensure equitable access and ethical governance of digital tools:** Invest in data-sharing systems, equitable AI tools, and transparent oversight to enable a shift from reactive crisis response to proactive disease prevention.

#### 4.5.2. Multisectoral One Health Perspective

Dengue’s drivers extend beyond healthcare, requiring coordination across sectors. By integrating diverse disciplines and sectors, the One Health model improves public health outcomes by addressing the root causes of dengue transmission and promoting a more holistic approach to disease prevention and control.

Key recommendations:

- **Institutionalize cross-sector collaboration:** Develop policies that formalize coordination and resource sharing among health, agriculture, environment, and urban planning sectors.
- **Support integrated pilot projects:** Launch and evaluate multisectoral demonstration initiatives to generate scalable models of integrated dengue control.
- **Engage communities as partners:** Promote sustained behavior change through public awareness campaigns, community mobilization, and citizen-led vector surveillance.
- **Implement climate-smart environmental strategies**: Reduce reliance on chemical insecticides and adapt vector control approaches to address climate-driven shifts in mosquito populations.

### 4.6. Community Engagement, Equity, and Risk Communication

Finally, reflecting the equity gaps and community-level challenges identified in Section 3.7 and Section 3.8, this section outlines strategies to strengthen community engagement, reduce inequities, and improve risk communication. Systemic disparities in access to dengue prevention and care must be addressed to reduce disease burden in vulnerable populations and ensure that interventions are both acceptable and effective. Community-centered strategies should prioritize those most at risk while strengthening trust in public institutions and health services.

Recommended actions include:

- **Strengthen primary care networks:** Build frontline health service capacity to identify and manage dengue in high-risk settings.
- **Target underserved communities:** Prioritize early diagnosis and treatment in informal settlements and low-income areas through subsidized services and mobile outreach. Strategies should also promote active community participation in prevention and care, supported by strategic communication approaches and the use of mHealth tools to strengthen active surveillance and encourage timely healthcare seeking.
- **Tailor outreach to population needs:** Use culturally appropriate messaging and gender-sensitive approaches, such as integrating dengue care into maternal-child health programs, to reach underserved groups effectively.

Limitations

- This work is based on a narrative, non-systematic review and expert deliberation, which presents several limitations. The literature search, using multiple databases and institutional sources, did not follow a registered protocol or formal systematic-review methods, potentially missing relevant publications, especially gray literature and non-indexed local data. Expert participants were chosen through professional networks, introducing potential selection bias and possibly failing to represent all perspectives from dengue-endemic countries in Latin America, particularly those of frontline community organizations and under-resourced subnational jurisdictions. Additionally, many data sources for situational analysis rely on passive surveillance systems, modeled estimates, or context-specific program evaluations, which are affected by underreporting, heterogeneous diagnostic capacity, and variability in health information systems across countries. Consequently, the generalizability of some findings and proposed strategies may be limited, and their applicability will depend on local epidemiological, legal, financial, and governance contexts. However, bringing together multidisciplinary experts from across Latin America allowed for the integration of diverse clinical, public health, regulatory, and community-level perspectives, enhancing the practical relevance of the recommendations for real-world decision-making. Finally, the rapidly evolving landscape of dengue epidemiology, climate-related risks, and vaccine and vector control technologies may render some evidence reviewed obsolete after the expert meeting and literature search; ongoing monitoring and context-specific evaluation will be essential to refine and adapt these recommendations over time, with the framework presented in this review serving as a foundation for that iterative process.

## 5. Conclusions

Integrated dengue control in Latin America faces persistent challenges, including underreporting, insecticide resistance, structural weakness in health systems, fragmented surveillance, financial and political constraints, and socioeconomic drivers. Overcoming these barriers requires a comprehensive, coordinated response.

A multifaceted strategy should prioritize expanding diagnostic capacity, adopting updated clinical protocols, ensuring equitable access to vaccination, using innovative vector control tools, and promoting cross-sectoral collaboration under a One Health approach, alongside targeted interventions to reduce health inequities. Incorporating the new-generation dengue vaccines into these efforts is justified by their demonstrated efficacy across diverse populations, providing an additional layer of protection in high-transmission settings. While national contexts differ, regional partnerships can help align efforts and share best practices without losing sight of local epidemiological realities.

Sustained progress will also depend on building stronger regional coordination mechanisms that allow countries to act collectively and position dengue control as a regional public good. This aligns with the growing emphasis on regional governance as a key level for epidemic preparedness and response in global health.

By aligning actions with timelines and stakeholders, this framework ensures dengue control evolves from crisis response to sustainable prevention, centering equity and multisectoral collaboration. In practical terms, recommendations can be prioritized within a timeframe based on feasibility and relative impact. Table 6 complements Figure 1 by detailing phased actions and responsible stakeholders.

Table 6 should be read as an actionable framework rather than a checklist of independent activities, with each phase building on the institutional capacity, surveillance data, clinical preparedness, and community trust accumulated in the preceding one, and with responsibility shifting progressively from health-sector actors in the short term to vector control, urban, and climate authorities in the medium term and to governments, international agencies, and research entities in the long term.

Operationalizing this framework requires anticipating several implementation challenges: limited municipal technical and financial capacity, inconsistent political prioritization between epidemic peaks, widespread insecticide resistance and other biological constraints, fragile interoperability across information systems, and uneven community trust in vector control and vaccination programs. Because epidemiological and ecological conditions vary substantially across Latin America—from dense urban centers and peri-urban informal settlements to indigenous and remote rural communities and high-mobility cross-border corridors—local health authorities are encouraged to adapt the framework to their context by adjusting the mix and intensity of interventions to local serotype circulation, vector ecology, housing and water storage practices, insecticide resistance profiles, and governance capacity. Success hinges on political will, funding, and iterative evaluation to adapt to emerging challenges like climate change and urbanization.

The evidence-based recommendations of AHF’s Stop Dengue Task Force offer a practical roadmap to strengthen integrated dengue prevention and control in Latin America, grounded in surveillance, guided by science, and centered on equity.

## Figures and Tables

**Table 1:** Key gaps and barriers to integrated dengue control in Latin America, by domain.

Domain	Specific Gap or Barrier	Main Adverse Outcome	Illustrative Example
Surveillance and information systems	Underreporting and limited laboratory confirmation	Underestimation of dengue burden and delayed outbreak detection	Passive surveillance, weak enforcement of mandatory reporting
	Fragmented and non-interoperable data platforms	Slow analysis and uncoordinated response	Parallel systems across Ministries; limited cross-border data sharing
Vector prevention and control	Widespread insecticide resistances	Reduced effectiveness of chemical control and persistent transmission	High resistance to permethrin in *Aedes aegypti* in Brazil, Colombia, and Mexico
	Inconsistent entomological surveillance	Poor identification of hotspots and misdirected interventions	Irregular Breteau and House index monitoring; limited geospatial mapping
Vaccines and treatment availability	Restricted indications and variable vaccine performance	Limited population-level protection	Dengvaxia restricted to seropositive individuals; gaps in access to newer vaccines
Health systems	Weak primary care and hospital surge capacity	Delayed diagnosis and treatment; excess morbidity and mortality	Overloaded emergency services during outbreaks; limited triage capacity
Cross-cutting and structural	Political and financial instability	Cyclical, reactive dengue control	Short-term outbreak funding; shifting priorities across political cycles
	Socioeconomic and environmental vulnerabilities	Concentration of dengue in marginalized populations	Informal settlements; inadequate water and sanitation infrastructure

**Table 2:** Factors contributing to inadequate dengue case detection in passive national surveillance systems.

Factor Category	Specific Factor	How It Impairs Case Detection
Healthcare-seeking behavior	Mild or asymptomatic infections	Individuals do not seek care’ large proportion of infections remain unreported
	Delayed consultation for febrile illness	Cases enter the system late, limiting early warning and timely response
Sociodemographic	Under recognition of illness in older adults	Lower consultation rates and missed diagnoses in a high-risk group
Clinical recognitions	Overlap with other febrile diseases	Misclassification as influenza, COVID-19, malaria, and other infections, leading to missed dengue notifications
Diagnostic capacity	Limited access to laboratory testing	Few cases confirmed
Legal and regulatory	Weak enforcement of mandatory reporting	Incomplete notification from public and private providers, especially in low-resource areas

**Table 3:** Comparison of dengue vaccines relevant to Latin America: indications, efficacy, and key considerations.

Feature	Dengvaxia (CYD-TDV)	TAK-003 (Qdenga)	Butantan-DV
Manufacturer and type	Sanofi Pasteur; recombinant tetravalent live-attenuated	Takeda; recombinant tetravalent live-attenuated	Instituto Butantan, Brazil; single-dose live-attenuated tetravalent
Schedule	3 doses (0, 6, 12 months)	2 doses (0, 3 months)	Single dose
Indicated population	Seropositive individuals only	Ages 6–16 years in high-transmission settings (WHO SAGE)	Approved in Brazil for ages 12–59 years
Reported efficacy	Variable across serotypes; reduces disease in seropositives	≅80% efficacy against virologically confirmed dengue at 12 months and 90% efficacy against hospitalization at 18 months	≅65% overall efficacy against symptomatic disease and 81% protection against severe dengue and hospitalization over 5 years of follow-up
Key safety considerations and limitations	Seronegatives: Higher risk of severe dengue; manufacture to cease by 2026	Seronegatives: Efficacy against DENV-3 not demonstrated; insufficient data for DENV-4	Approved use limited to Brazil; regional regulatory pathways still expanding
References	[49,50,51]	[52,53]	[54,55]

**Table 4:** Key Monitoring Indicators Across Surveillance Domains.

Monitoring Domain	Key Indicators	Operational Use
Epidemiological [16,17,18]	Incidence rates, weekly case counts, hospitalization and mortality stratified by age and sex, and case geographic clustering	Identifies abnormal case spikes relative to historical baselines and supports targeted, hotspot-driven interventions
Entomological [37,38,39]	House Index, Breteau Index, Container Index, and adult female *Aedes aegypti* density from larval surveys and adult-trapping methods	Assesses vector abundance and breeding efficacy to direct source-reduction campaigns and inform the timing of insecticide applications
Climatic and environmental [32,81,82,83]	Precipitation, temperature, humidity, El Niño/La Niña, and vegetation indices	Anticipates ecological conditions favorable to mosquito breeding
Non-traditional and digital surveillance proxies [17,19]	Care-seeking patterns for febrile illness, hospital admissions, and digital signals (e.g., internet-media and digital epidemiology platforms)	Serves as an early proxy for rising community transmission
Laboratory	Predominant circulating serotypes serotype shifts over time, and viral diagnostic outputs [35,36]	Anticipates clinical severity; introduction or re-emergence of a serotype is associated with elevated risk of severe disease and outbreak intensification [23]

**Table 5:** Integrated vector control approaches and implementation strategies for dengue prevention.

Strategy	Operational Value
Wolbachia-infected mosquito deployments	Sustainable reduction in dengue transmission potential through suppression of vector competence in endemic urban settings [45]
Attractive toxic sugar baits (ATSBs)	Complementary vector control strategy targeting adult mosquito populations in urban and peri-urban environments [39]
Spatial repellents and emanators	Complementary indoor protection strategies that reduce mosquito-human contact in semi-enclosed environments [39]
Autocidal gravid ovitraps (AGO)	Combined surveillance and vector control approach targeting gravid female mosquitoes [84]
Precision larval source management	Targeted identification and treatment of highly productive breeding sites using entomological and environmental data [85]
Integrated vector management (IVM)	Coordinated framework integrating biological, environmental, technological, and community-based interventions according to local epidemiological risk [86]
Environmental management and community engagement	Sustainable reduction of mosquito-breeding sites through improved water storage, drainage, waste management, and community participation [84,85]

**Table 6:** Timelines and Stakeholder responsibilities for implementation of Task Force recommendations.

Time Horizon	Key Actions	Responsible Stakeholders
0–2 years	Enhance diagnostics; update clinical protocols; improve surveillance; commence vaccination; strengthen community-based activities	Ministries of Health; healthcare providers; community leaders
3–5 years	Scale vector control innovations; develop predictive models; institutionalize multisectoral approaches; build regional capacity; address inequities	Vector and urban authorities; climate agencies; regional bodies
6+ years	Establish resilient systems; foster research and development; embed dengue in policy and governance; conduct impact assessments	Governments; international agencies; research entities

## Data Availability

No new data were created or analyzed in this study. Data sharing is not applicable to this article.

## Abbreviations

The following abbreviations are used in this manuscript:

AHF	Americas Health Foundation
ATSB	Attractive Toxic Sugar Baits
DVI	Dengue Vulnerability Indices
EHR	Electronic health records
GIS	Geospatial information systems
IVM	Integrated Vector Management
PAHO	Pan-American Health Organization
POCT	Point-of-care testing
WHO	World Health Organization
